# Comparative risk factors for nosocomial New Delhi metallo-beta-lactamase (NDM-1) producing Enterobacteriaceae and non-carbapenemase producing (NCP) Enterobacteriaceae

**DOI:** 10.1186/2047-2994-4-S1-P134

**Published:** 2015-06-16

**Authors:** E Izharuddin, K Marimuthu, P Bee Fong, K Jia Qi, A Chow, DC Lye, P Krishnan, B Ang

**Affiliations:** 1 Infectious Disease, Tan Tock Seng Hospital, Singapore; 2 Infection Control, Tan Tock Seng Hospital, Singapore; 3 Laboratory Medicine, Tan Tock Seng Hospital, Singapore

## Introduction

Carbapenem resistance occurs via two mechanisms: acquisition of resistance genes (NDM-1, KPC, OXA) or combination of extended spectrum beta-lactamase production and alteration of the expression of porins (NCP *Enterobacteriaceae*).

## Objectives

We conducted a case-case-control study to identify if NDM-1 *Enterobacteriaceae* share risk factors with NCP *Enterobacteriaceae*.

## Methods

Patients admitted for at least 48 hours between September 2010 and July 2013 were included. Case 1: Patients with NDM-1 *Enterobacteriaceae* isolated from either clinical or surveillance cultures. Case 2: Patients with NCP *Enterobacteriaceae* isolated from either clinical or surveillance cultures. Control: Patients screened negative for carbapenem-resistant *Enterobacteriaceae* (CRE). Patients with both NDM-1 *Enterobacteriaceae* and NCP *Enterobacteriaceae* were excluded. Demographic, clinical, microbiological and antibiotics usage data were collected from electronic medical records. We conducted time at risk adjusted bivariate analysis followed by multivariate analysis using STATA 12.0.

## Results

A total of 1934 patients were screened for CRE. A total of 40 NDM-1 *Enterobacteriaceae* and 43 NCP *Enterobacteriaceae* patients were compared with 61 randomly selected control patients. There was no significant difference in age, sex and Charlson index between cases and controls. Independent risk factor for NDM-1 *Enterobacteriaceae* was intensive care unit (ICU) admission in the preceding 3 months (OR, 3.2; 95% CI 1.2 – 8.6; p=0.02) and for NCP *Enterobacteriaceae* was number of days exposed to carbapenems (OR 1.2; 95% CI 1.1 – 1.3; p=0.002).

## Conclusion

NDM-1 *Enterobacteriaceae* and NCP *Enterobacteriaceae* do not share similar risk factors in this small single center study. This finding has an implication on infection control strategies for CRE control.

## Disclosure of interest

None declared.

